# Association between individual quadriceps muscle volume/enthesis and patello femoral joint cartilage morphology

**DOI:** 10.1186/ar4426

**Published:** 2014-01-01

**Authors:** Hechmi Toumi, Thomas M Best, Marija Mazor, Raphael Coursier, Antonio Pinti, Eric Lespessailles

**Affiliations:** 1 EA4708 Orleans University, IPROS, CHRO, 1, rue Porte-Madeleine, BP 2439, 45032 Orleans, cedex 1, France; 2 Division of Sports Medicine, Department of Family Medicine, Sports Health and Performance Institute, The Ohio State University, Columbus, OH 43221, USA; 3 Groupement des Hôpitaux de l’Institut Catholique de Lille (GHICL)/Faculté Libre de Médecine, F-59000 Lille, France; 4 Département de traumatologie-orthopédie France, UCLille, Lille, France

## Abstract

**Introduction:**

The aim of this study was to determine the association between individual quadriceps muscle volumes and the quadriceps enthesis structures and cartilage morphology at the patellofemoral joint (PFJ).

**Methods:**

We studied 12 cadavers (age 75 ± 5 years). For both legs, individual quadriceps muscles (vastus lateralis (VL), rectus femoris (RF), vastus intermedialis (VI) and vastus medialis (VM)) were dissected and their volumes measured. Cartilage areas at the PFJ were classified using the International Cartilage Repair Society (ICRS) score. Histological sections were evaluated at the quadriceps tendon enthesis (laterally, centrally and medially). Several variables were calculated on the binary images based on two-dimensional analysis. These were apparent bone area (BA) and apparent trabecular thickness (TH). A Spearman rank test was used to determine the strength of correlation between individual quadriceps muscles volume, the structure of the quadriceps tendon enthesis and the ICRS score.

**Results:**

The thickness of calcified fibrocartilage tissue was significantly greater in the central part of the enthesis than both medially (*P* = 0.03) and laterally (*P* = 0.04). Uncalcified fibrocartilage was significantly thicker laterally (*P* = 0.04) and centrally (*P* = 0.02) than medially. Muscle volume was highest (*P* <0.05) for the VL, followed by the VI, VM and RF. There was no association between total and individual muscle volumes and ICRS or BA. However, there was a strong positive correlation (r = 0.81) between the VL/VM volume ratio and BA ratio (bone volume at the lateral part divided by bone volume at the medial part). There was a moderate positive correlation between VL/VM and ICRS (r = 0.65) and between ICRS and BA ratio (lateral/medial; r = 0.74).

**Conclusions:**

Individual and total quadriceps volumes were not correlated with cartilage loss at the PFJ or fibrocartilage thickness. However, both VL/VM and BA ratio (lateral/medial) were positively correlated with ICRS scoring and therefore could be a tool for predicting degree of PFJ osteoarthritis severity.

## Introduction

Radiographic osteoarthritis (OA) of the knee affects more than 33% of people age 60 years and older [1]. Recently, it has been shown that 13.7% of the subjects (age 48 to 58 years) had radiographic knee OA (Kellgren/Lawrence grade ≥2) in at least one knee, and the prevalence increased to 47.8% by year 15 (age 63 to 73 years) [2]. To optimize the management of OA, it is important to increase our knowledge regarding predictors for progression of this common and disabling condition. One topic that has been explored in recent years is the role of the quadriceps in patellofemoral pain and knee OA [3,4]. Magnetic resonance imaging (MRI) is often used in studying the relationship between muscle cross-sectional area (CSA) and the risk for patella femoral joint (PFJ) OA [3-5]. It appears that the vastus lateralis (VL) and vastus medialis (VM) cross-sectional areas are positively associated with thickness of patellar cartilage and bone volumes [3-5]. Furthermore, it has been suggested that subjects with a greater VL/VM CSA ratio have fewer radiographic findings associated with knee OA [4]. However, a limitation of this study is the fact that muscle segmentations were derived from a single slice of the quadriceps, thereby not necessarily being an accurate representation of the geometry of the entire muscle [4]. To our knowledge, there are no data which have reported an association between trabecular bone microarchitecture/quadriceps enthesis and knee OA.

The analysis of trabecular bone microarchitecture provides valuable information on stress patterns within cancellous bone [6]. Movement and load are the mechanical stimuli that trigger the metaplasia of fibroblasts to fibrocartilage cells [7]. There is a good correlation between the quantity of uncalcified fibrocartilage at an enthesis and the extent of movement that occurs between tendon/ligament and bone [7]. Therefore, to estimate the amount of load transmitted by each individual quadriceps muscle, we propose to analyze the fibrocartilaginous structure of the quadriceps tendon enthesis at the sites where each individual muscle is attached.

The aim of the present study was to investigate the impact of individual quadriceps muscles, and their volume ratios, on the quadriceps tendon enthesis/patella structures and the related association with PFJ OA. Our hypothesis was that changes in patellofemoral joint morphology would correlate with quadriceps muscle volume. Such information will help to clarify the contribution of each individual muscle to the load transferred to the patella and its association with PFJ OA. Our long-term goal is to utilize such information to determine if targeted interventions aimed at the specific muscles can be utilized to mitigate the disabling features of progressive OA of the PFJ.

## Methods

The study was approved by the NHS Auvergne Ethics Research Committee (agreement number GHK/MM1425) and was conducted in accordance with the principles of the Declaration of Helsinki. Twelve dissecting room fresh cadavers (less than 48 hours after death, six male and six female) were utilized. They were anonymous voluntary donations and consent forms were provided by the department of Anatomy at the Faculty of Medicine and approved by the committee listed above. Donor age ranged from 68 to 82 years at the time of death (75 ± 5 mean +/- SD years) (Table 1). Details about the lifetime activities of the donors were not available; however, none of the donors’ knees appeared to have undergone lower limb operation procedures. Both quadriceps tendon enthesis from each cadaver were used for cartilage, histological and histomorphometrical analysis.

**Table 1:** Donors age, individual and total quadriceps femoris muscles volume

	**Age**	**VM**	**RF**	**VI**	**VL**	**Total**
Average	75 ± 5	152 ± 22	78 ± 17	204 ± 16	247 ± 24	682 ± 21
Male	76 ± 3	175 ± 18	81 ± 12	220 ± 28	272 ± 21	750 ± 23
Female	74 ± 4	129 ± 19	74 ± 14	187 ± 27	223 ± 19	616 ± 22

Values are means ± SD in cm^3^. RF, rectus femoris; VI, vastus intermedialis; VL, vastus lateralis; VM, vastus medialis.

### Anthropometric analysis of the quadriceps muscles

To estimate muscle volume for the four different quadriceps muscles, specimens were collected from both legs. Careful layer-by-layer dissection included separation of the four parts of the quadriceps tendon as far as possible without destroying existing decussations between the tendons of the individual muscles. The tendinous fibers of the four muscles were followed as far as possible toward their bony insertions. Each muscle was then placed in a 5 L graduated cylinder filled with tap water. Muscle volume was calculated as the difference in the pre- and post volume measurement. Measurements were performed independently by two blinded technicians.

### Cartilage evaluation

Cartilage areas at the anterior surface of the trochlea and patella were classified by the International Cartilage Repair Society (ICRS) scoring system [8]. Overall repair assessment was scored and later graded as follows: grade 0, normal cartilage; grade 1, near normal cartilage with superficial lesions; grade 2, cartilage with lesions extending to <50% of the depth of the cartilage; grade 3, cartilage with defects that extend to >50% of the depth of the cartilage; and grade 4, severely abnormal cartilage in which the cartilage defects reach subchondral bone [8]. When multiple cartilage defects were observed the highest grade was scored. Grading was performed independently by two blinded surgeons. In cases where grading was different between the two evaluators, a third surgeon was called to resolve the difference.

### Histological analysis

**Figure 1:**
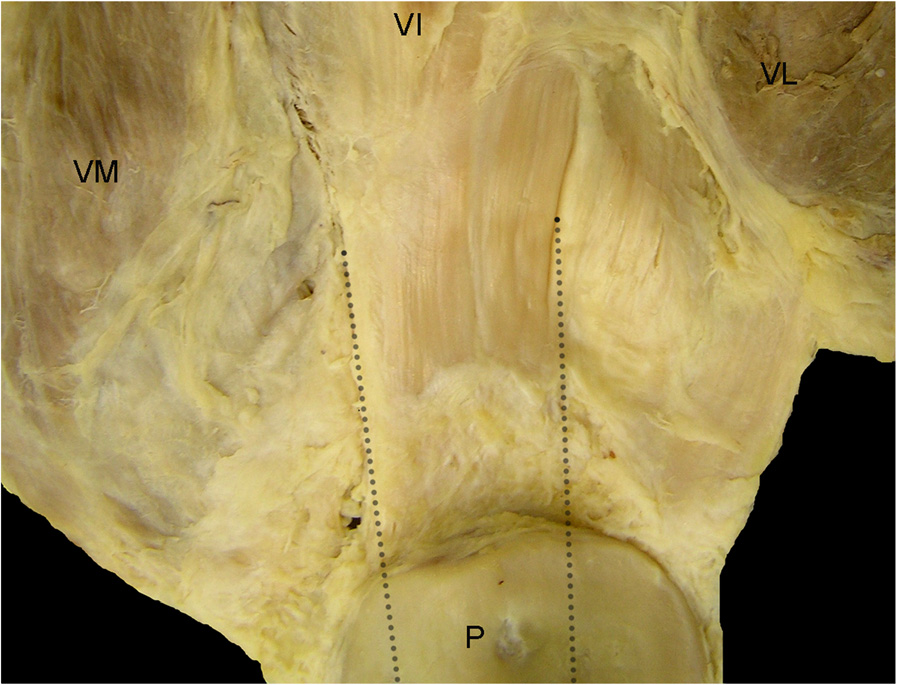
**Gross anatomy of the patella and the femoral quadriceps: posterior views.** VM, vastus medialis; VI, vatsus intermedialis; VL, vastus lateralis; and P, patella. Dotted lines correspond to the longitudinal cuts which were then continued through the patella itself dividing the patella into medial, central and lateral parts.

The procedures used have been described in a previous study [9]. Briefly, the quadriceps tendon enthesis was removed by cutting the patellae transversally in the middle and the quadriceps tendon approximately 1 cm from the bone. The patella was placed with the posterior side uppermost; the three parts of the quadriceps tendon (lateral part: VL, central part: rectus femoris (RF) + vastus intermedialis (VI) and medial part: VM) were carefully dissected layer-by-layer as close to the patella as possible. The longitudinal cuts were then continued through the patella itself dividing the patella into medial, central and lateral parts (Figure 1). For examining the quadriceps tendon enthesis, tissue was post-fixed in 10% neutral buffered formalin, decalcified with 5% nitric acid, dehydrated through a graded alcohol series, cleared in xylene and embedded in paraffin wax. Serial longitudinal sections were cut at 8 μm throughout the medial, central and lateral thirds of the enthesis and 12 sections were mounted on glass slides at 1-mm intervals. Slides were stained with Hall and Brunt’s quadruple stain, Masson’s trichrome (for photography) and toluidine blue (for fibrocartilage metachromasia).

### Trabeculae structure analysis

The same protocol used in the present study has been developed and previously validated [9]. Histological sections were evaluated with an Epson scanner (Epson perfection 1250 Model G820A, Japan) and a high-resolution algorithm (1200 * 2400 dpi). Imaging parameters were identical for all slides. Images were transferred to a personal computer and structural 146 analyses of the bone were performed. Algorithms used to characterize bone architecture were all developed using Matlab Software. The analyses used have been described previously in [9]. The boundary between the thin cortical bone and the underlying trabecular bone was defined using an automatic contour detection algorithm. Subsequently, a segmentation process permitted separation of spicules from bone marrow. The segmentation was made with an edge detection using a LaPlacian–Gaussian filter that included both a smoothing filter (which convolutes the image by a Gaussian filter) and a second-order derivative filter. Tuning of this combined filter addresses the size of the smoothing window, but also the variance of the convolutive Gaussian filter. Zero-crossing detection in the resulting image provides a binary image in which dark regions represent the bone marrow and light regions represent trabeculae [9]. Several variables were calculated on the binary images based on two-dimensional analysis. These were apparent bone area (BA) = trabecular bone/tissue volume and (TH) apparent trabecular thickness (TH) = 2/(trabecular separation/apparent bone area) [10].

### Morphometric analysis of histological sections

For all three parts (medial, central and lateral) of the enthesis and following a method developed by Evans *et al*. [11], the thickness of total calcified fibrocartilage tissue (CF) was determined by measuring the thickness of the cortical zone of calcified tissue (calcified cartilage and lamellar bone). The thickness of the zone of uncalcified fibrocartilage (UF) was estimated by measuring the distance from the tidemark to the furthest recognizable chondrocyte within the tendon. Five such measurements were taken for each of the three regions (medial, central and lateral) at equal intervals across the attachment site on one slide at each 1-mm sample point. Mean values and standard deviations were then used for statistical comparisons.

### Statistical analysis

Five variables were quantified for muscle volume; total quadriceps volume (VI + VM + RF + VL) along with each muscle contribution (individual muscle volume divided by the total quadriceps volume expressed in %). Four variables for the bone and enthesis structures (UF, CF, BA and TH) measured at three different sites (lateral, central and medial) were also quantified. The Kolmogorov-Smirnov test was used to access data normality. As the data were not normally distributed, a non-parametric statistical test (Wilcoxon) was used to compare the different variables with an *a priori* significance level of *P* = 0.05 accepted. Since a comparison of the structure of the different sides of the patella enthesis was of primary interest, we also calculated the lateral to medial ratio for each variable. Variable correlation was assessed using the Spearman test. Correlations were considered very strong when the r value was above 0.8, moderate between 0.60 and 0.79, fair between 0.30 and 0.59 and weak when below 0.29 [10].

## Results

**Figure 2:**
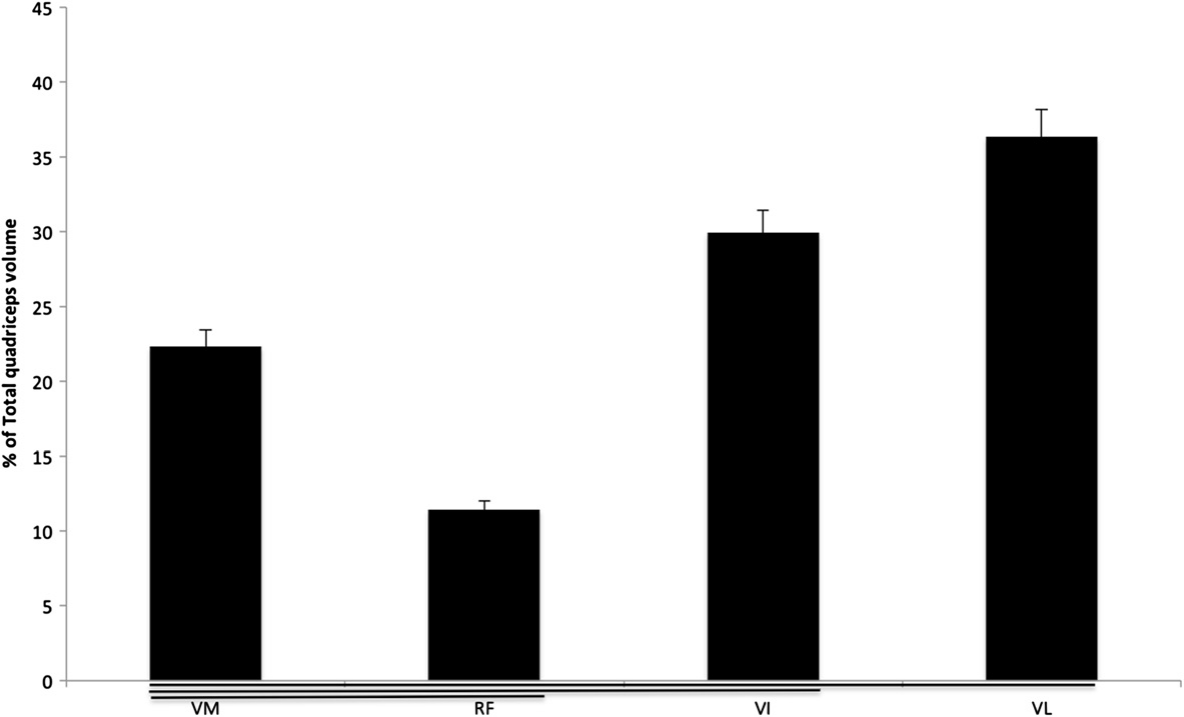
**Individual muscle volume expressed as a percentage of total quadriceps volume.** Values are means ± SD. Significant differences (*P* < 0.05) are underlined.

There were significant differences in muscle volume between the four individual quadriceps components; 157 ± 17, 78 ± 14, 204 ± 21 and 248 ± 19 cm^3^ for the VM, RF, VI and VL, respectively. Values for each muscle contribution to the total quadriceps volume were 22.3% ± 3.1, 11.4% ± 2.3, 29.9% ± 2.1 and 36.3% ± 3.8 for the VM, RF, VI and VL, respectively (Figure 2). Men presented a significantly higher (*P* = 0.02) total quadriceps volume compared to women (750 ± 23 cm^3^ for men and 616 ± 22 cm^3^ for women) (Table 1). The differences in muscle volume values between men and women were 26.2% ± 3.9%, 9% ± 2.1, 15.3% 19 ± 2.8 and 18.1% ± 3.3, respectively, for VM, VI, RF and VL.

**Figure 3:**
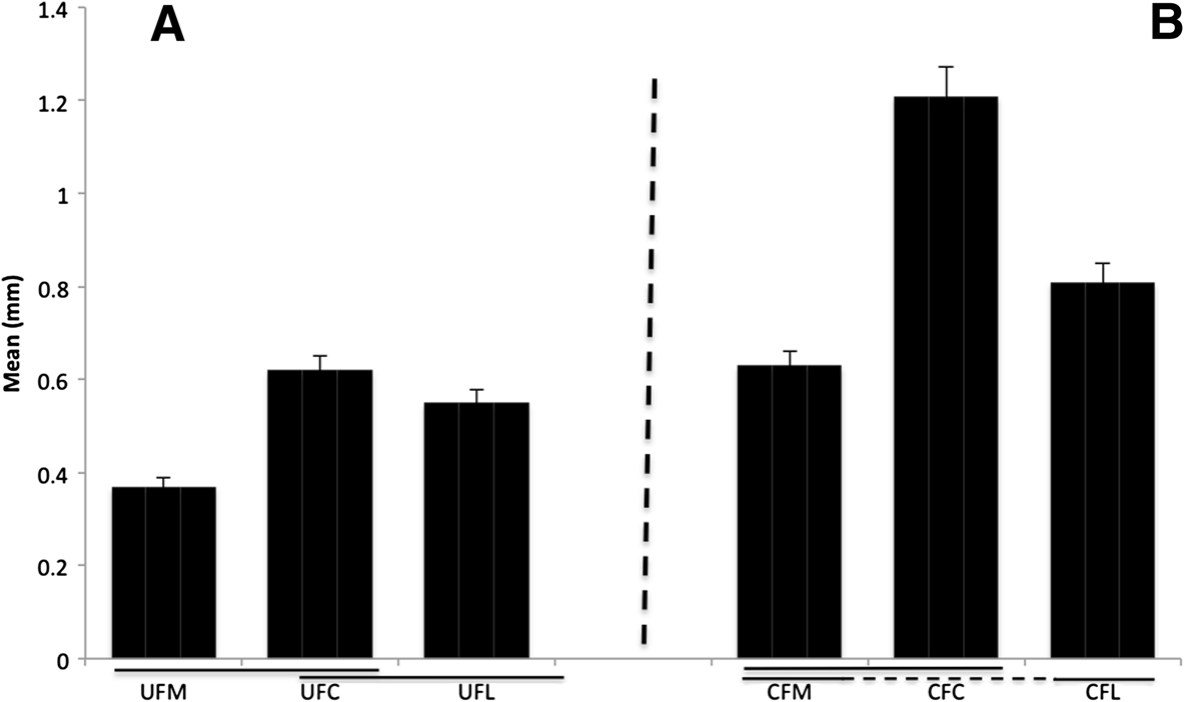
**Fibrocartilage thickness at the quadriceps tendon enthesis. (A)** calcified fibrocartilage, **(B)** uncalcified fibrocartilage. Values are means ± SD. Significant differences (*P* < 0.05) are underlined.

The thickness of the cortical zone of calcified fibrocartilage tissue (calcified cartilage and lamellar bone) was significantly greater in the central part of the enthesis than it was both medially (*P* = 0.03) and laterally (*P* = 0.04) (Figure 3A). Values were 1.21 ± 0.14 mm centrally, 0.81 ± 0.17 mm laterally and 0.63 ± 0.21 mm medially. There was also a significant difference between the medial and lateral side with the thickness of the calcified tissue higher laterally (*P* = 0.04). The zone of uncalcified fibrocartilage was significantly thicker laterally and centrally than medially (Figure 3B). Values were 0.62 ± 0.15 mm centrally, 0.55 ± 0.06 mm laterally and 0.37 ± 0.09 mm medially. However, no significant difference was observed between the central and lateral parts (*P* = 0.1).

**Figure 4:**
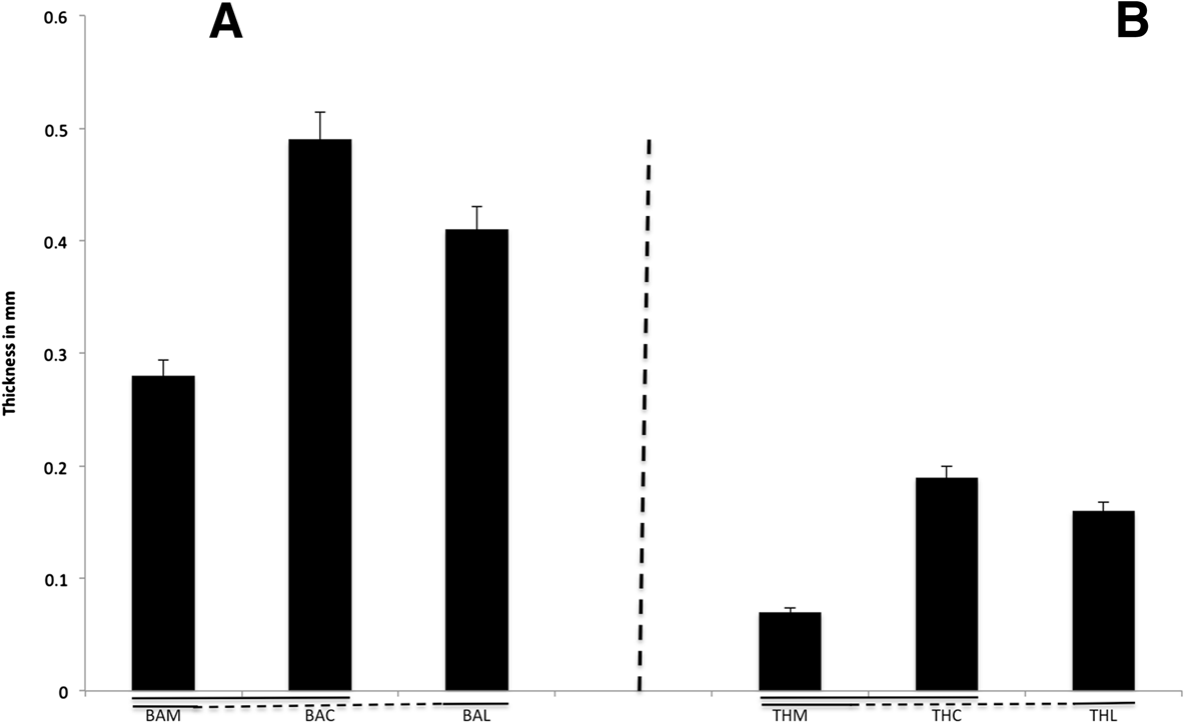
**Apparent bone area and trabeculea thickness at the quadriceps tendon enthesis. (A)** Apparent bone area in the lateral (BAL), central (BAC) and medial (BAM) patella facets expressed as a percentage of the total tissue volume (mean values ± SD). Significant differences (*P* < 0.05) are underlined. Values are significantly higher centrally and laterally than medially. **(B)** Trabeculae thickness in the lateral (THL), central (THC) and medial (THM) patella facets expressed as a percentage of the total tissue volume (mean values ± SD). Significant differences (*P* < 0.05) are underlined. Values are significantly higher centrally and laterally than medially.

Bone structure analysis is shown in Figure 4. The BA (corresponding to the sum of trabecular volume divided by the total tissue volume) in the proximal region of the patella was significantly higher centrally and laterally than it was medially (*P* = 0.01, *P* = 0.03, respectively) (that is, central: 0.49 ± 0.06, lateral: 0.41 ± 0.04, medial: 0.28 ± 0.07, Figure 4A). There were similar differences in the apparent TH (Figure 4B). The trabeculae were thicker in the central and lateral parts compared to the medial region (that is, central: 0.19 ± 0.06 mm, lateral: 0.16 ± 0.05 mm, medial: 0.07 ± 0.03 mm, *P* = 0.02, *P* = 0.04 respectively).

### Association of individual muscle volume with enthesis structure (CF, UFC, BA and TH)

There was a strong positive correlation between the central and lateral parts of the quadriceps enthesis for the CF, BA and TH (r = 0.89, r = 0.92 and r = 0.90, respectively). There was a fair positive correlation (r = 0.31, *P* = 0.03) between ICRS and VL, fair inverse correlation (r = -0.32, *P* = 0.04) between ICRS and VM and a fair positive correlation (r = 0.29, *P* = 0.03) between ICRS and quadriceps total volume. The correlations of all other variables were weak (below 0.29).

The lateral to medial ratio for each variable is shown in Table 2. There were several strong and moderately positive correlations when the Spearman test was applied to the lateral/medial ratio values (Table 2). Strong positive correlations were found between VL/VM and UF ratio (r = 0.8, *P* = 0.02) and between VL/VM and BA ratio (r = 0.81, *P* = 0.02). In addition, there were moderately positive correlations between: (1) ICRS and VL/VM (r = 0.65, P = 0.03); (2) ICRS and BA ratio (lateral/medial) (r = 0.74, *P* = 0.02); and (3) ICRS and TH ratio (r = 0.67, *P* = 0.03).

**Table 2:** Correlation coefficient values from the Spearman test applied to the lateral/medial ratio values

	**VL/VM**	**UF**	**CF**	**BA**	**TH**	**ICRS**
VL/VM	1	0.80^a^	0.72^b^	0.81^a^	0.56	0.65
UF	0.80^a^	1	0.64^b^	0.69^b^	0.52	0.49
CF	0.72^b^	0.64^b^	1	0.55	0.49	0.41
BA	0.81^a^	0.69	0.55	1	0.78^b^	0.74^b^
TH	0.56	0.52	0.49	0.78^b^	1	0.67^b^
ICRS	0.65	0.49	0.41	0.74^b^	0.67^b^	1

^a^Very strong (above 0.8); ^b^moderate between 0.60 to 0.79. BA, apparent bone area; CF, calcified fibrocartilage tissue; ICRS, International Cartilage Repair Society; TH, apparent trabecular thickness; UF, uncalcified fibrocartilage; VL, vastus lateralis; VM, vastus medialis.

## Discussion

Osteoarthritis is the most common joint disorder typically presenting symptoms first in middle age adults and often leading to significant impairment in function and mobility. Studies have been conducted recently to automatically segment the quadriceps muscles in order to aid clinicians in their study of the development, causes and effects of osteoarthritis [3-5]. It has previously been observed that VM cross-sectional area, based on a single slice MRI measurement, is positively associated with patellar cartilage and bone volumes in healthy subjects [3]. Findings from the present study confirm a strong positive correlation between VL/VM volume and both BA ratio (lateral/medial) and ICRS grade. In the present study, the VL/VM ratio was 1.63 and the ICRS average was 2.35 ± 0.55 (cartilage with defects that extend to >50% of the depth of the cartilage). Previously, [12] observed a VL/VM ratio of 1.33 from a population of subjects with knee OA between 51 and 71 years old. On the other hand, [13] noted a VL/VM ratio of 1.22 in a population of similar age but no history of arthritis or neuromuscular problems. Together, these findings may suggest that the VL/VM ratio is higher in patients with PFJ arthritis compared to a similar age-matched population with no history of knee OA. However, additional studies will be needed to determine causality given the retrospective nature of the current work. The clinical significance of this observation remains unknown but may represent an area of further investigation based on targeted interventions to modify muscle geometries and symptoms of knee OA.

Our histological analysis revealed regional variation in the fibrocartilage at the quadriceps tendon enthesis and the trabeculae architecture of the proximal region of the patella. Collectively, the data indicated that there was a greater amount of fibrocartilage at the quadriceps tendon enthesis centrally and laterally than medially. Interestingly, this pattern is consistent with the muscle volume findings we noted. Both the lateral and central parts of the quadriceps (VL and VI) had a higher volume than the medial portion of the quadriceps (VM). A higher muscle volume laterally and centrally can theoretically produce higher muscle forces, which may explain why more fibrocartilage was observed at these regions compared to the medial side. Surprisingly, the correlation between muscle volume and PFJ OA was not strong. There was only a fair positive correlation (r = 0.31) between the ICRS and the volume of the VL, a fair negative correlation (r = -0.32) with VM and a weak positive correlation (r = 0.29) with quadriceps total volume. Previous studies [14] based on MRI analysis found that muscle CSAs of the thigh display a higher correlation with cartilage volume than basic anthropometric variables. However, it is important to mention that the correlation coefficients with cartilage thickness were also either fair or weak. Values were +0.44, +0.35 and +0.24, respectively, for muscle CSAs, body height and weight. There were, however, strong positive correlations between VL/VM and both UF ratio (lateral/medial) and BA ratio (lateral/medial). To perhaps explain these associations, it is important to understand the enthesis concept and, in particular, how the patella reacts to the unbalanced load transmitted from the quadriceps muscles group. The concept of a ‘functional enthesis’ serves to emphasize anatomical, biomechanical and pathological features that are shared between a true fibrocartilaginous enthesis and regions proximal to the attachment sites themselves where tendons or ligaments wrap around bony pulleys [15]. Gao and Messner [16] have suggested that the tensile loads to which a ligament is subjected determine the shape and surface area of the calcified fibrocartilage-bone interface. This is in line with the earlier suggestion of [11] that differences in the thickness of the subchondral plate at the enthesis are related to regional variations in tensile loading. Our data demonstrate that fibrocartilage thickness does not correlate with total quadriceps and individual muscle volumes. However, there was a positive correlation between fibrocartilage thickness and the VL/VM volume ratio, which suggests that it is not the amount of load transferred to the enthesis that promotes fibrocartilage and bone remodeling, but it is the load balance and direction of loading. This is in accordance with a previous finding where the greatest quantity of fibrocartilage was observed in the deepest part of the attachment (proximal); it is related to the extent to which each tendon is free to move near its enthesis and not to the amount of load applied. A previous investigation noted that the most mobile tendon has the greatest thickness of fibrocartilage [17].

### Limitations

Our results should be viewed in light of several limitations. First, this study was conducted on 12 cadaveric subjects (both legs were used) with an average age of 75 ± 5 and the ICRS average was 2.35 ± 0.55. Second, this study is retrospective. Nevertheless, our findings do suggest that muscle ratio rather than individual muscle analysis may provide insights into the possibility that muscle volumes do indeed predict cartilage morphology.

## Conclusions

Our study demonstrates that ICRS scoring is positively correlated with the BA ratio (lateral/medial) and VL/VM. Although the aim of the study was not to investigate if there was any variance between MRI results and similar data obtained directly from sample tissues, the parallel findings obtained in relation to the correlation between VL/VM ratio and OA suggests that the MRI limitations (evaluation of muscle size using CSA instead of muscle volume and not excluding the fat tissue) have no significant effect on the overall correlation between VL/VM ratio and knee OA. To the best of our knowledge, this is the first study which has measured two independent parameters (individual muscle volume and enthesis structure of each individual muscle) to estimate individual quadriceps muscle contribution to the PFJ.

## Abbreviations

BA: Apparent bone area; CF: Calcified fibrocartilage tissue; CSA: Cross-sectional area; ICRS: International Cartilage Repair Society; MRI: Magnetic resonance imaging; OA: Radiographic osteoarthritis; PFJ: Patella femoral joint; RF: Rectus femoris; TH: Apparent trabecular thickness; UF: Uncalcified fibrocartilage; VI: Vastus intermedialis; VL: Vastus lateralis; VM: Vastus medialis.

## Competing interests

The authors declare that they have no competing interests.

## Authors’ contributions

HT, TB and EL designed the study/protocol. HT, MM, RC and AP carried out the experiments. HT, TB and AP performed the statistical analysis. HT, EL and TB coordinated and drafted the manuscript. All authors read and approved the final manuscript.
